# Impact of Brief Lactation Rotation in Residency on Decision to Refer for Lactation Support

**DOI:** 10.1177/21501319241298751

**Published:** 2024-11-07

**Authors:** Megan M. Kindred, Kelsie R. Barta

**Affiliations:** 1 The University of Texas Health Science Center at Houston, Houston, TX, USA; 2 University of Houston, Houston, TX, USA

**Keywords:** breastfeeding, medical education, referral, multidisciplinary care team, primary health care

## Abstract

**Introduction:**

Breastfeeding challenges may be difficult to address within the constraints of a typical well-child visit. A team-based approach, including lactation consultants, can reduce physician workload, increase breastfeeding self-efficacy, and increase patient satisfaction. Continuity of care issues, including absent or delayed referrals, hinder patient access to skilled lactation support.

**Objectives:**

To examine the post-residency referral patterns of physicians to lactation consultants among physicians who did or did not participate in a brief outpatient lactation rotation at an academic lactation clinic during residency.

**Methods:**

A cross-sectional online survey of physicians who completed residency programs in pediatrics, family medicine, or medicine-pediatrics between 2013 and 2022 was conducted using REDCap; 46 valid responses were received.

**Results:**

Respondents who participated in a brief outpatient lactation rotation during residency refer patients to lactation consultants with significantly higher frequency than those who did not complete the rotation. Among those with lactation consultants in their geographic area, this relationship remained significant even when adjusting for residency type, gender, experience with an inpatient lactation consultant, and infant feeding attitudes using multiple linear regression.

**Conclusions:**

An outpatient lactation rotation during medical residency may increase the likelihood of referring to a lactation consultant in post-residency practice, which can improve breastfeeding outcomes.

## Introduction

Despite the well-documented benefits of breastfeeding, physicians have historically reported that they receive little education about breastfeeding.^1^ While education surrounding human lactation in medical education and residency programs has increased, more than 50% of physicians report that their breastfeeding training in residency was inadequate, and only approximately half rate themselves as effective at providing breastfeeding counseling.^2^ Additionally, most indicated a lack of time to counsel families about breastfeeding and a desire for additional interactive breastfeeding education.^1-3^

Assisting with breastfeeding difficulties is time-intensive and may require several follow-up appointments before problems are fully resolved. Lactation consultants often work with patients in recurrent appointments lasting 1 to 2 hours to address lactation problems. Due to time constraints, many lactation-related issues cannot be thoroughly addressed by physicians in a typical well-child visit. Physicians may find it helpful to refer patients with lactation-related issues to lactation consultants in the same way they would refer a patient with a speech or motor disorder to a speech-language pathologist, occupational therapist, or physical therapist. In the Surgeon General’s Call to Action to Support Breastfeeding, physicians are encouraged to utilize a team approach to support those having trouble breastfeeding, and it explicitly states that this team should include an International Board Certified Lactation Consultant (IBCLC).^4^ However, some research suggests that challenges with collaboration can hinder patient access to skilled lactation support. A systematic review of qualitative studies identified lack of referrals and delayed referrals as barriers to providing appropriate breastfeeding support.^5^ Similarly, in a recent survey of lactation support providers in Appalachia, 84.3% identified challenges related to other health professionals as a barrier influencing their ability to provide lactation support.^6^ Although prior research has shown that lack of continuity of care is a barrier to coordinated breastfeeding support, no research has been done to assess the impact of breastfeeding-related residency training on physician decisions to refer to lactation consultants.^7^

Because lactation consultants may help reduce physician workload, improve parental breastfeeding self-efficacy, and increase patient satisfaction, it is important to determine factors that affect physician decisions to refer to lactation consultants.^8,9^ In addition to providing medical residents with breastfeeding education, outpatient rotations with lactation consultants familiarize physicians with the lactation consultant’s role beyond the hospital setting and may increase referrals to outpatient lactation consultants in clinical practice. Therefore, the objective of this study was to examine the post-residency referral patterns of physicians to lactation consultants among former pediatric, family medicine, and medicine-pediatrics residents who did or did not participate in a 4- to 8-hour outpatient lactation rotation at an urban academic lactation clinic during residency.

## Methods

### Study Design, Setting, and Participants

A cross-sectional observational survey design was used for this study, conducted at an academic medical center in a major Texas city. Data collection occurred during the 6 weeks from October 9, 2023, to November 20, 2023. Participants were physicians who were eligible for inclusion if they (a) completed medical residency at a pediatrics, family medicine, or combined medicine-pediatrics program that routinely has residents participate in a brief outpatient lactation rotation, (b) graduated from residency from 2013 to 2022, and (c) were currently practicing medicine in the United States. Participants who indicated that they never care for breastfeeding patients were excluded from the study.

**Figure 1.**
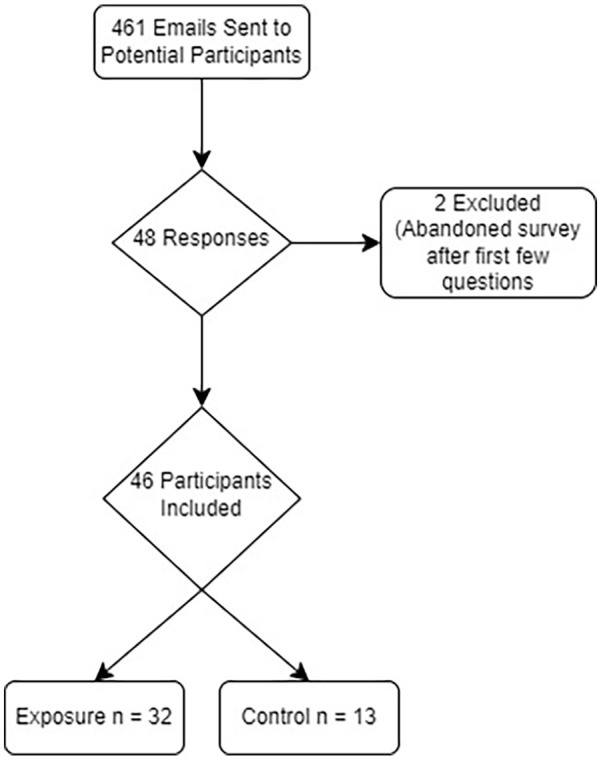
Participant Flow Diagram.

Emails were sent to 461 residency graduates inviting them to complete an anonymous online survey in REDCap.^10^ A reminder email was sent 1 week following the initial survey invitation, and the survey was programmed to accept responses for 6 weeks. An a priori power analysis was conducted using G*Power version 3.1.9.7 to determine the minimal sample size needed for our analyses.^11^ With 80% power, an alpha level of .05, and 5 predictors, at least 55 responses were required to detect a medium effect (*f*^2^ = 0.15) or at least 25 responses to detect a large effect (*f*^2^ = 0.35) in a multivariable linear regression model. Therefore, our goal was to have a response rate of at least 11.9% to achieve 55 total responses. A participant flow diagram illustrating the number of individuals who received the questionnaire, those who completed it or were excluded, and the final distribution of participants in the exposure and control groups is provided in Figure 1.

### Exposure and Control Groups

Resident physicians completed brief outpatient lactation rotations in 1 to 2 4-hour blocks at an academic outpatient breastfeeding clinic staffed by board-certified lactation consultants. Rotations typically consisted of basic breastfeeding education and observation of lactation clinic visits. Clinic visits were conducted by lactation consultants in 75 to 90-minute intervals. A typical visit included a history and assessment of the lactating parent and breastfeeding infant and a pre-feed and post-feed weight measurement to assess overall growth and milk transfer from parent to infant. Shared decision-making principles were used to develop a post-visit plan for parents to implement at home.

Although many residents in the collaborating residency programs were able to attend the outpatient lactation rotation, some residents were reassigned to cover other services on the days they were scheduled for the rotation or were absent due to illness or vacation. Others completed residency in a year that their program did not send residents to complete the rotation. In this study, the residents who did not complete the outpatient lactation rotation served as the control group, while the residents who completed the rotation comprised the exposure group.

### Survey Items

The primary outcome of this study was lactation referral frequency, which was assessed by self-report. Respondents were asked: “For your patients who are struggling with breastfeeding, how often do you recommend that they get breastfeeding assistance from a lactation consultant?” Response options ranged from 0 to 4 where 0 = *never* and 4 = *always*. Respondents were only asked this question if they answered yes when asked, “In your current practice setting, do you ever assess or obtain information about how breastfeeding is going?” as those who do not ever assess breastfeeding would not have the need to refer to a lactation consultant.

Demographic information, including gender, race/ethnicity, and birth year, was obtained from respondents through self-report (see supplementary file). Additional variables were included because of their theoretical potential to influence referral frequency. These included how frequently respondents care for breastfeeding patients (1 = *very rarely* to 5 = *very often*), whether there are lactation consultants available to support breastfeeding in the geographic region in which the respondent practices, whether they had any other clinical education experience with a lactation consultant (in the hospital, in a patient’s home, or at another outpatient clinic), and infant feeding attitudes operationalized through the 17-item Iowa Infant Feeding Attitude Scale (IIFAS).^12^

The IIFAS is used to measure maternal attitudes toward infant feeding and has demonstrated reliability and validity in a wide variety of populations.^12^ Items responses are selected on a 5-point scale (1 = *strongly disagree*, 5 = *strongly agree*) with 9 items reverse scored. Scores range from 17 to 85, with higher scores indicating a preference for breastfeeding. In initial psychometric testing, the Cronbach’s alpha of the IIFAS ranged from .85 to .86, indicating excellent reliability.^13^ Although the IIFAS was originally designed to be used in maternal populations, it has demonstrated acceptable reliability in other populations, including males aged 21 to 44 in the United States (α = .78),^14^ undergraduate nursing students (α = .74),^15^ and medical students (α = .77).^16^ In this study’s sample, the Cronbach alpha coefficient for the IIFAS was .74.

### Statistical Analysis

Descriptive statistics are reported for all variables, including means and standard deviations for normally distributed variables and medians and interquartile ranges for non-normal variables. For each variable, differences between the exposure and control groups were assessed using independent samples *t* tests, Chi square tests, or Fisher exact tests.

For all variables except the IIFAS score, cases were excluded in an analysis when there was missing data for an included variable (see Table 1). For the IIFAS score, responses with no more than 1 missing item were retained. For these responses, the value of the missing item was imputed using maximum likelihood estimation (expectation-maximization algorithm), and the score for the 17 scale items was subsequently calculated.^13^

**Table 1.** Differences Between Respondents Completing and Not Completing Lactation Clinic Rotation.

Characteristic	Total respondents (*n* = 46)^a^	Respondents grouped by lactation clinic rotation		Test statistic with *P* value
		No rotation (*n* = 13)	Rotation (*n* = 32)	
Age, mean (SD), year^b^	34.9 (3.0)	36 (2.9)	34.4 (3.0)	*t* = 1.6, *df* = 42, *P* = .121
Year of residency completion, mean (SD)^b^	2019 (2.7)	2018 (2.6)	2019 (2.6)	*t* = −1.6, *df* = 41, *P* = .128
IIFAS score, mean (SD)^b^	64.2 (7.1)	63.4 (7.4)	64.6 (7.2)	*t* = −0.44, *df* = 37, *P* = .660
Female, no. (%)^b^	33 (73.3)	7 (53.8)	26 (81.3)	*X*^2^ = 3.6, *df* = 1, *P* = .060
Race/ethnicity, no. (%)				*P* = .115^c^
White, not Hispanic	19 (41.3)	4 (30.8)	15 (46.9)	
Black	3 (6.5)	2 (15.4)	1 (3.1)	
Asian	14 (30.4)	3 (23.1)	11 (34.4)	
Hispanic	8 (17.4)	2 (15.4)	5 (15.6)	
Other or more than 1 race	2 (4.3)	2 (15.4)	0 (0)	
Residency, no. (%)				*P* < .001^c^
Family medicine	17 (37)	10 (76.9)	7 (21.9)	
Pediatrics	27 (58.7)	2 (15.4)	24 (75)	
Medicine-pediatrics	2 (4.3)	1 (7.7)	1 (3.1)	
Frequency caring for BFG parent or infant, No. (%)				*P* = .405^c^
Very rarely	6 (13)	3 (23.1)	3 (9.4)	
Rarely	2 (4.3)	0 (0)	2 (6.3)	
Occasionally	12 (26.1)	5 (38.5)	6 (18.8)	
Somewhat often	6 (13)	1 (7.7)	5 (15.6)	
Very often	20 (43.5)	4 (30.8)	16 (50)	
Practice setting, no. (%)^d^				
Primary care	32 (69.6)	11 (84.6)	20 (62.5)	*P* = .178^c^
Emergency medicine or urgent care	2 (4.3)	0 (0)	2 (6.3)	*P* = 1.00^c^
Hospitalist	7 (15.2)	2 (15.4)	5 (15.6)	*P* = 1.00^c^
Subspecialty	8 (17.4)	2 (15.4)	6 (18.8)	*P* = 1.00^c^
Still in training	4 (8.7)	0 (0)	4 (12.5)	*P* = .308^c^
Other	1 (2.2)	0 (0)	1 (3.1)	*P* = 1.00^c^
Assesses how BFG is going, no. (%)	34 (73.9)	9 (69.2)	24 (75)	*P* = .721^c^
LC in geographic region, no. (%)^b^				*P* = .166^c^
Yes	37 (80.4)	8 (66.7)	28 (87.5)	
No	5 (10.9)	3 (25)	2 (6.3)	
Unsure	3 (6.5)	1 (8.3)	2 (6.3)	
Any other LC experience, no. (%)	25 (54.3)	6 (46.2)	18 (56.3)	*X*^2^ = 0.38, *df* = 1, *P* = .538

Abbreviations: BFG, breastfeeding; IIFAS, Iowa Infant Feeding Attitude Scale; LC, lactation consultant.

a One respondent unsure of lactation rotation completion.

b Missing values: age, 1; IIFAS, 6; year of residency completion, 2; gender, 1; LC in geographic region, 1.

c Fisher’s exact.

d Respondents able to select more than 1 response option.

An initial bivariate analysis was completed to determine if there was a significant difference in the frequency of referring patients who are struggling with breastfeeding to a lactation consultant between the exposure and control groups. Due to the ordinal nature of the outcome variable, a Mann-Whitney U test was initially used to assess the difference between the 2 groups. Then, a multivariable model adjusted for the potential impact of those variables with bivariate differences between groups at *P* values ≤.10. In this model, we also adjusted for IIFAS score, the presence of a lactation consultant in the physician’s geographic area of practice, and participation in other lactation rotations based on the theoretical likelihood that these variables may have significantly impacted the frequency of referral to lactation consultants.

The primary multivariable model evaluated the relationship between the completion of the brief outpatient lactation rotation and reported referral frequency using multivariable linear regression. Although the outcome variable is ordinal, it was entered in the linear regression model here as an ordinal approximation of a continuous variable. Linear regression was chosen as the primary model over ordinal regression for 2 reasons. First, because linear regression is robust, ordinal variables with 5 or more categories can be used as continuous variables without introducing significant bias.^17,18^ Second, linear regression models are interpreted in a more straightforward manner than ordinal regression models. Backward stepwise elimination was used to select variables for the final regression model. Variables with *P* values >.10 were removed, starting with the variable with the highest *P* value and ending when all variables in the model had *P* values ≤.10. A sensitivity analysis was conducted using a multivariable ordinal regression model, including the same variables in the final linear regression model. A subgroup analysis was conducted with responses from participants who indicated that there is a lactation consultant available within their geographic region of practice using both multivariable linear and ordinal regression models. SPSS version 29 was used for data analysis.

## Results

### Respondent Characteristics

Forty-eight survey responses were received. Two were excluded because the respondent answered only the first few questions before abandoning the survey. Ultimately, 46 responses were included in the analysis, a 10% response rate. Respondent characteristics are displayed in Table 1. Most of the respondents were female (72.7%), White (41.3%) or Asian (30.4%), graduates of pediatrics residency programs (58.7%), and currently practicing in a primary care setting (69.6%). Nearly half (44%) indicated that they care for breastfeeding parents or infants very often, and more than 80% indicated that there is a lactation consultant available within their region of practice.

Comparing those who completed the brief outpatient lactation rotation with those who did not, there was a significant difference based on residency type, with significantly more former pediatrics residents (92%) completing the rotation compared to former family medicine residents (41%). A higher percentage of females (79%) than males (50%) completed the brief outpatient lactation rotation. There were no significant differences in other experiences with lactation consultants or IIFAS scores between the 2 groups.

The mean IIFAS score was 64.2 ± 7.1, indicating the sample had neutral attitudes toward breastfeeding overall. About 72% of respondents who completed the IIFAS had scores corresponding to neutral breastfeeding attitudes (IIFAS scores 49–69), and the remainder (28%) had scores corresponding to positive attitudes toward breastfeeding (IIFAS scores 70–85). No respondents had an IIFAS score that indicated negative attitudes toward breastfeeding.

### Impact of Brief Outpatient Lactation Rotation on Physician Referrals to Lactation Consultants

An initial bivariate analysis was conducted to assess the relationship between the completion of the lactation rotation and the decision to refer to lactation consultants, and a significant difference was identified. Respondents who completed the lactation rotation reported significantly higher frequency of referring to lactation consultants than those who did not, *U* = 164, *P* = .023.

To further evaluate this relationship, a multivariable linear regression was used to adjust for potential covariates. Gender, residency type, presence of lactation consultant in the respondent’s geographic area of practice, completion of any other rotations with lactation consultants, and IIFAS score were entered into the baseline model based on differences between exposure and control groups and theoretical impact on the outcome of interest. Following backward stepwise elimination, the final model was constructed to include rotation completion and to adjust for IIFAS scale score and presence of a lactation consultant in the physician’s geographic region of practice (Table 2).

**Table 2.** Multivariable Linear Regression for Frequency of Physician Referral to Lactation Consultants.

Variables	*B*	SE: *B*	*t*	β	95% CI *B*		*P*
					LL	UL	
Constant	4.100	1.468	2.793		1.064	7.136	.010
IIFAS score	−0.052	0.022	−2.376	−.320	−0.097	−0.007	.026
LC in geographic area of practice	1.972	0.424	4.652	.596	1.095	2.849	<.001
Outpatient lactation rotation	1.091	0.338	3.231	.436	0.392	1.789	.004
	*R*^2^ = .629, *R*^2^_adjusted_ = .580, *F*(3, 23) = 12.99						<.001

Abbreviations: IIFAS, Iowa Infant Feeding Attitude Scale; LC, lactation consultant.

The final linear regression model (*n* = 27) evaluating the relationship between completion of a brief outpatient lactation rotation and frequency of referring to a lactation consultant was significant when adjusting for IIFAS score and presence of a lactation consultant in the respondent’s geographic area of practice (Table 2). Those who completed the lactation rotation were, on average, 1 level (never, rarely, sometimes, usually, always) more likely to refer to a lactation consultant than those who did not complete the rotation when IIFAS score and presence of a lactation consultant in the respondent’s geographic area of practice were held constant (*B* = 1.091, *t* = 3.231, *P* = .004). IIFAS score was negatively associated with frequency of physician referral to a lactation consultant such that a 20-point increase in IIFAS score is associated with a 1 level decrease in frequency of referral (*B* = −0.052, *t* = −2.376, *P* = .026).

We conducted a sensitivity analysis using a multivariable ordinal regression (*n* = 27; complementary log-log link) with the same 3 predictors (Table 3).^19,20^ The predictors accounted for a significant amount of variance in the frequency of referring to a lactation consultant. The odds of those completing the lactation rotation being more frequent referrers in post-residency practice were 8.3 (95% CI, 1.8 to 37.6) times that of those who did not complete the rotation. Every 1-point increase in IIFAS score was associated with a 10% decrease in the odds of being a more frequent referrer. The 3 predictors accounted for approximately 74% of the variance in the frequency of referral to a lactation consultant.

**Table 3.** Multivariable Ordinal Logistic Regression for Frequency of Physician Referral to Lactation Consultants.

Variables	*B*	SE: *B*	OR	95% CI OR		*P*
				LL	UL	
IIFAS score	−0.101	0.050	0.904	0.819	0.997	.044
LC in geographic area of practice						
No	Reference					
Yes	3.674	1.191	39.4	3.82	407.1	.002
Outpatient lactation rotation						
No	Reference					
Yes	2.111	0.773	8.26	1.81	37.6	.006
McFadden’s pseudo-*R*^2^ = .717, Likelihood ratio *X*^2^(3) = 45.09, complementary log-log link						<.001

Abbreviations: IIFAS, Iowa Infant Feeding Attitude Scale; LC, lactation consultant.

In a subgroup analysis of those indicating that there is a lactation consultant available in their geographic area (*n* = 24), only completion of the lactation rotation was significantly associated with the frequency of referral to a lactation consultant in both the multivariable linear and ordinal regression models (Tables 4 and 5). IIFAS score was not significant in either model.

**Table 4.** Multivariable Linear Regression for Frequency of Physician Referral to Lactation Consultants when Lactation Consultant is Available in Geographic Area.

Variables	*B*	SE: *B*	*t*	β	95% CI *B*		*P*
					LL	UL	
Constant	4.626	1.378	3.356		1.760	7.493	.003
IIFAS score	−0.032	0.021	−1.501	−.257	−0.077	0.012	.148
Outpatient lactation rotation	1.267	0.323	3.928	.674	0.596	1.938	<.001
	*R*^2^ = .427, *R*^2^_adjusted_ = .372, *F*(2, 21) = 7.82						.003

Abbreviations: IIFAS, Iowa Infant Feeding Attitude Scale.

**Table 5.** Multivariable Ordinal Logistic Regression for Frequency of Physician Referral to Lactation Consultants when Lactation Consultant is Available in Geographic Area.

Variables	*B*	SE: *B*	OR	95% CI OR		*P*
				LL	UL	
IIFAS score	−0.086	0.059	0.918	0.818	1.030	.145
Outpatient lactation rotation						
No	Reference					
Yes	2.688	0.919	14.70	2.43	89.03	.003
	McFadden’s pseudo-*R*^2^ = .194, Likelihood ratio *X*^2^(2) = 8.54, complementary log-log link					.014

Abbreviations: IIFAS, Iowa Infant Feeding Attitude Scale.

## Discussion

In all models, completion of the brief outpatient lactation rotation was significantly associated with a higher frequency of referring patients experiencing breastfeeding difficulties to a lactation consultant. This study provides preliminary evidence that outpatient lactation rotations in residency may increase referrals by physicians to lactation consultants. Although there are no prior studies linking outpatient lactation rotations in residency with future referral patterns, other studies have found an increase in referrals to a specialty among physicians who completed a rotation in that specialty.^21,22^ It may be that these rotations increase physician familiarity with the specialty and provide knowledge that helps physicians identify patients who would benefit from referral.

Evaluating patient utilization of lactation services following physician referrals to lactation consultants exceeded the scope of this study. However, other evidence supports the assumption that increased referrals can improve access to skilled lactation support. In research on cardiac rehabilitation, a strong physician recommendation was a key independent predictor of cardiac rehabilitation participation.^23^ Similarly, in a quasi-experimental study of well-child care physician referrals of infants for preventative dental care, both active and passive referrals increased the odds of having a dental visit in the first year of life, with active referrals having a larger effect.^24^ In an integrative review, the advice, preferences, and practices of health professionals have been identified as factors influencing women’s infant feeding decisions.^25^

In some of the models, infant feeding attitudes were related to referrals in an unexpected direction, with higher IIFAS scores indicative of more positive attitudes toward breastfeeding decreasing the odds of being a frequent referrer. However, in the subsample of participants who reported that lactation consultants were available in their geographic location, infant feeding attitudes were not associated with referral patterns. Physicians without ready access to lactation consultants in their region are less likely to be frequent referrers for apparent reasons: there is either no one available to refer to or a referral would require patients to travel further to see a lactation consultant. It may be that these physicians have more positive attitudes toward breastfeeding if they are more directly involved in the care of their patients due to a lack of referral options. Given the study’s sample size and small effect size of IIFAS in the models, future research is needed to clarify the relationships between physician attitudes toward infant feeding and patterns of referral to lactation consultants.

The sampling for this study introduces the possibility of several types of bias. While an email was distributed to every physician who completed residency from each of the 3 residency programs between 2012 and 2023, participation was voluntary. Self-selection bias is inherent in this approach, as those who choose to complete a survey may differ from those who do not. In our case, participants may have been more likely to participate if they had stronger feelings about breastfeeding or breastfeeding education and less likely to participate if they were more ambivalent on these topics. Additionally, social desirability bias may have been a factor. Recruitment emails were sent from the first author’s email address and identified the sender as the director of the outpatient lactation clinic in which exposure group participants completed their rotations. Given the stated goals of the research and the researcher’s identity, it is possible participants responded in a way that they felt would be more desirable to the researchers. To reduce the risk of this type of bias, questions were worded neutrally, and response options included multi-step scales rather than a dichotomous yes or no.

Another significant limitation of this study is the response rate of 10%, which falls below our a priori target based on power analysis, raising further concerns about representativeness and non-response bias. Although we sampled the entire eligible population of residency graduates and sent a reminder email to enhance participation, only 46 responses were ultimately usable. Nevertheless, our analyses achieved statistical significance, indicating that the findings remain robust within the context of the responses received.

## Conclusions

In our study, an outpatient lactation rotation during physician resident training increased the frequency of post-residency referrals to lactation consultants. Including diverse breastfeeding education experiences in physician resident training can increase patient access to lactation support through its integration with other healthcare services. Future research is needed to examine the relationship between breastfeeding attitudes, lactation education, and physician referrals.

## Supplemental Material

sj-pdf-1-jpc-10.1177_21501319241298751 – Supplemental material for Impact of Brief Lactation Rotation in Residency on Decision to Refer for Lactation Support Supplemental material, sj-pdf-1-jpc-10.1177_21501319241298751 for Impact of Brief Lactation Rotation in Residency on Decision to Refer for Lactation Support by Megan M. Kindred and Kelsie R. Barta in Journal of Primary Care & Community Health
