# Supplementary material for: Impact of Brief Lactation Rotation in Residency on Decision to Refer for Lactation Support
Source: J Prim Care Community Health. 2024 Nov 7;15:21501319241298751. doi: 10.1177/21501319241298751 (PMC11544676; doi:10.1177/21501319241298751)
Supplement: sj-pdf-1-jpc-10.1177_21501319241298751 – Supplemental material for Impact of Brief Lactation Rotation in Residency on Decision to Refer for Lactation Support [file sj-pdf-1-jpc-10.1177_21501319241298751.pdf]

Residency Lactation Education Survey

We acknowledge that not all lactating people consider themselves mothers or identify as women and may prefer to use terms other than breastfeeding or breast milk. For the sake of brevity, we refer to breastfeeding and breast milk intending to encompass all forms of human milk feeding whether at the breast or chest or by other feeding device. Additionally, to maintain the language and validity of the original Iowa Infant Feeding Scale, we have preserved the use of the words mother, breastfeed, breast milk, and their variations within the scale.

For this survey, breastfeeding patients include either or both members of the breastfeeding dyad:

(a) lactating parents who are breastfeeding or pumping breast milk

(b) infants or children who are breastfeeding or being fed breast milk.

Which residency program did you attend?

- ◯ UTHealth Houston Pediatrics residency
- ◯ UTHealth Houston Med-Peds residency
- ◯ UTHealth Houston Family Medicine residency
- ◯ Memorial Family Medicine residency
- ◯ Other residency program

Are you currently practicing medicine in the United States?

- ◯ Yes
- ◯ No

	Never	Very rarely	Rarely	Occasionally	Somewhat often	Very often
How often do you care for patients who are breastfeeding?	◯	◯	◯	◯	◯	◯

During residency, did you complete a rotation at the Lactation Foundation?

The Lactation Foundation is the breastfeeding clinic associated with McGovern Medical School at UTHealth Houston. Rotations are typically 1-2 days that are 4-8 hours each.

- ◯ Yes
- ◯ No
- ◯ Unsure

What is your gender?

- ◯ Female
- ◯ Male
- ◯ Other, please specify ______

Are you Hispanic, Latino/a, or Spanish origin?

- ◯ Yes
- ◯ No

What is your race?
(One or more categories may be selected.)

- ☐ White
- ☐ Black or African American
- ☐ Asian
- ☐ American Indian or Alaska Native
- ☐ Native Hawaiian or Other Pacific Islander
- ☐ Other

What year were you born?
(Please enter the 4-digit year of your birth)

____

What year did you complete the [residency]?

(Please enter the 4-digit year.)

____

What is your current practice setting?<br>(One or more categories may be selected.)	☐ Primary care outpatient<br>☐ Urgent care or emergency medicine<br>☐ Hospitalist<br>☐ Subspecialty<br>☐ Still in training<br>☐ Other, please specify ______
Do you hold the International Board Certified Lactation Consultant (IBCLC) certification?	◯ Yes<br>◯ No
Are there lactation consultants available to support breastfeeding in the geographic region in which you care for patients?	◯ Yes<br>◯ No<br>◯ Unsure

For the following question, if you do not assess or obtain information about how breastfeeding is going, please select "not applicable."

	Not applicable	Never	Rarely	Sometimes	Usually	Always
For your patients who are struggling with breastfeeding, how often do you recommend that they get breastfeeding assistance from a lactation consultant?	○	○	○	○	○	○

Which breastfeeding topics were or would have been most helpful to learn about during your residency?

(One or more topics may be selected.)

- ☐ Benefits of breastfeeding
- ☐ Current breastfeeding recommendations
- ☐ Physiology of milk production
- ☐ Assessing breastfeeding quality
- ☐ Assessing adequacy of breast milk intake
- ☐ Counseling patient/parent with low milk supply
- ☐ Counseling patient/parent with sore or damaged nipples
- ☐ Counseling parent of an infant who will not latch or is not latching well
- ☐ Counseling patient/parent about engorgement
- ☐ Counseling patient/parent on returning to work while breastfeeding
- ☐ Assessing, treating, and counseling patient/parent with mastitis
- ☐ Assessing latch
- ☐ Assessing infant oral anatomy
- ☐ Assessing infant suck mechanics
- ☐ Using and counseling patient/parent about nipple shields
- ☐ Managing breastfeeding infants with poor weight gain
- ☐ Prescribing medications to lactating patient/parent
- ☐ Other, please specify ______
- ☐ None

	not well at all				very well
How well did your residency program prepare you to counsel breastfeeding patients about breastfeeding-related topics?	○	○	○	○	○

For the following questions, if you do not assess or obtain information about how breastfeeding is going, please select "not applicable."

	Not applicable	Never	Rarely	Sometimes	Usually	Always
When I encounter a patient who is struggling with breastfeeding, I have enough knowledge to effectively help them.	◯	◯	◯	◯	◯	◯
When I encounter a patient who is struggling with breastfeeding, I have enough hands-on experience to effectively help them.	◯	◯	◯	◯	◯	◯
When I encounter a patient who is struggling with breastfeeding, I have enough time to effectively help them.	◯	◯	◯	◯	◯	◯

Other than your rotation at the Lactation Foundation, in which of the following settings did you have a clinical education experience with a lactation consultant during residency?
(One or more options may be selected.)

- ☐ in the hospital
- ☐ in a patient's home
- ☐ an outpatient clinic other than the Lactation Foundation
- ☐ other, please specify ______
- ☐ none of the above

During your residency, in which of the following settings did you have a clinical education experience with a lactation consultant?
(More than one option may be selected.)

- ☐ in the hospital
- ☐ in an outpatient clinic
- ☐ in a patient's home
- ☐ other, please specify ______
- ☐ none of the above

For each of the following statements, please indicate how much you agree or disagree.

	strongly disagree	disagree	neutral	agree	strongly agree
The nutritional benefits of breast milk last only until the baby is weaned from breast milk.	○	○	○	○	○
Formula feeding is more convenient than breastfeeding.	○	○	○	○	○
Breastfeeding increases mother-infant bonding.	○	○	○	○	○
Breast milk is lacking in iron.	○	○	○	○	○
Formula-fed babies are more likely to be overfed than are breastfed babies.	○	○	○	○	○
Formula feeding is the better choice if the mother plans to work outside the home.	○	○	○	○	○
Mothers who formula-feed miss one of the great joys of motherhood.	○	○	○	○	○
Women should not breastfeed in public places such as restaurants.	○	○	○	○	○
Babies fed breast milk are healthier than babies who are fed formula.	○	○	○	○	○
Breastfed babies are more likely to be overfed than formula-fed babies.	○	○	○	○	○
Fathers feel left out if a mother breastfeeds.	○	○	○	○	○
Breast milk is the ideal food for babies.	○	○	○	○	○
Breast milk is more easily digested than formula.	○	○	○	○	○
Formula is as healthy for an infant as breast milk.	○	○	○	○	○
Breastfeeding is more convenient than formula feeding.	○	○	○	○	○
Breast milk is less expensive than formula.	○	○	○	○	○
A mother who occasionally drinks alcohol should not breastfeed her baby.	○	○	○	○	○

If there is anything else you would like us to know about your experience with lactation education during residency, please tell us here. __________________________________
